# Effects of a footwear intervention on foot pain and disability in people with gout: a randomised controlled trial

**DOI:** 10.1186/s13075-019-1886-y

**Published:** 2019-04-24

**Authors:** Mike Frecklington, Nicola Dalbeth, Peter McNair, Trish Morpeth, Alain C. Vandal, Peter Gow, Keith Rome

**Affiliations:** 1 Health and Rehabilitation Research Institute, AUT University, Private Bag 92006, Auckland, 1142 New Zealand; 2 Department of Medicine, The University of Auckland, Auckland, New Zealand; 3 Auckland District Health Board, Auckland, New Zealand; 4 Department of Biostatistics & Epidemiology, AUT University, Auckland, New Zealand; 5 Research & Evaluation Office, Ko Awatea, Counties Manukau Health, Auckland, New Zealand; 6 Counties Manukau District Health Board, Auckland, New Zealand

**Keywords:** Gout, Foot, Footwear, Foot pain

## Abstract

**Background:**

There is limited evidence supporting the long-term effect of a foot care package that includes footwear for people with gout. The aim of this study was to investigate the effectiveness of a footwear intervention on foot pain and disability in people with gout over 6 months.

**Methods:**

Participants with gout (*n* = 94) were randomly allocated to either a control group (podiatric care and gout education) or footwear intervention group (podiatric care and gout education plus a commercially available athletic shoe). Measurements were undertaken at baseline and 2, 4, and 6 months. Primary outcome was foot pain severity. Secondary outcomes were overall pain, foot impairment/disability, footwear comfort, fit, ease and weight. Data were analysed using repeated measures models.

**Results:**

Baseline foot pain scores were low, and no differences in foot pain scores were observed between groups over 6 months (adjusted effect estimate: − 6.7, 95% CI − 16.4 to 2.9, *P* = 0.17). Improvements between groups in overall pain scores (adjusted effect estimate: − 13.2, 95% CI − 22.2 to − 4.3, *P* < 0.01) and foot impairment/disability scores (− 4.7, 95% CI − 9.1 to − 0.3, *P* = 0.04) favouring the footwear intervention were observed at 2 months, but not at 4 or 6 months. Improvements between groups in footwear fit (adjusted effect estimate: − 11.1, 95% CI − 21.1 to − 1.0, *P* = 0.03), ease (− 13.2, 95% CI − 23.8 to − 2.7, *P* = 0.01) and weight (− 10.3, 95% CI − 19.8 to − 0.8, *P* = 0.03) favouring the footwear intervention were also observed over 6 months. Similar improvements were observed for footwear comfort at 2 and 4 months. No other differences in secondary outcomes measured were observed at 6 months (*P* > 0.05).

**Conclusions:**

Addition of footwear to a foot care package did not improve foot pain in people with gout. Short-term improvements in overall pain and foot impairment/disability and more durable improvements in footwear comfort and fit were observed with the footwear intervention.

**Trial registration:**

ACTRN12614000209695. Registered 27 February 2014, http://www.anzctr.org.au/TrialSearch.aspx?searchTxt=ACTRN12614000209695&isBasic=True

**Electronic supplementary material:**

The online version of this article (10.1186/s13075-019-1886-y) contains supplementary material, which is available to authorized users.

## Background

Gout commonly affects the articular and soft tissue structures of the feet, especially the first metatarsophalangeal joint [1] and Achilles tendon [2]. Foot problems are commonly described by people with gout [3], and people with gout experience high levels of foot pain, impairment and disability [4].

Regular podiatric care is associated with a reduction in foot pain and disability in people with inflammatory arthritis, including those with gout [5]. Prescribing footwear may be part of a foot care package for people with arthritis affecting the foot and ankle. For example, footwear interventions can improve foot pain and function in people with rheumatoid arthritis and foot osteoarthritis [6]. In rheumatoid arthritis, footwear interventions can also improve plantar pressure measurements and walking speed [6].

A footwear intervention may also benefit people with gout. A substantial proportion of people with gout wear footwear lacking in cushioning, support, stability and motion control [7]. Furthermore, patients with gout wearing poor footwear report higher pain and disability scores [7]. We have reported the results of a feasibility study, showing that commercially available athletic footwear with heel and forefoot cushioning, a dual density midsole and rocker sole reduces foot pain and disability in people with gout at 2 months [8].

Currently, the evidence supporting the long-term effect of a foot care package that includes footwear for people with gout is limited. The aim of this study was to investigate whether addition of footwear to a foot care package has benefit on foot pain and disability in people with gout over 6 months.

## Methods

### Study design

The study was a 6-month, two-arm, parallel randomised controlled trial comparing two foot care packages for people with gout, registered as a clinical trial with the Australian New Zealand Clinical Trials Registry (ACTRN12614000209695).

### Participants

Participants were recruited from public hospital rheumatology clinics and through public newspaper advertising throughout Auckland, New Zealand. Participants were recruited between October 2014 and June 2016. Inclusion criteria were gout according to the 1977 preliminary American Rheumatism Association classification criteria [9] and over 20 years of age. Exclusion criteria were history of other inflammatory arthritis or neuromuscular disease, experiencing a gout flare at time of screening visit, medication for foot pain in the past 4 weeks, prescription of footwear and/or foot orthoses in the past 3 months, previous foot and ankle surgery or unable to walk 10 m unaided. The trial was approved by the New Zealand Ministry of Health, Health and Disability Ethics Committees (14/CEN/117), and all participants provided written informed consent.

### Randomisation and blinding

Participants were allocated 1:1 to the control group (podiatric care and gout education) or footwear intervention group (podiatric care and gout education plus a commercially available athletic shoe) using unstratified block randomisation with random block sizes. Centralised randomisation allowed the use of a sealed opaque envelope system. Randomisation of participants was undertaken by a research assistant with sole access to envelopes and not involved in data collection. Treating clinicians were not involved in the randomisation of participants. Participants could not be blinded to their study group. Participants invited into the study were informed they would receive a foot care package, without specific mention of footwear. Post-randomisation, participants were not informed of the intervention modalities in the other randomisation group.

### Assessment

Participants attended study visits at the Auckland University of Technology Podiatry Clinic from November 2014 to February 2017. Baseline assessment included the recording of age, gender, ethnicity, body mass index (BMI), medical history and current medications. Disease-specific data included latest serum urate, disease duration, number of gout flares in the last 3 months and tophus count (total and at the foot).

### Interventions

**Fig. 1:**
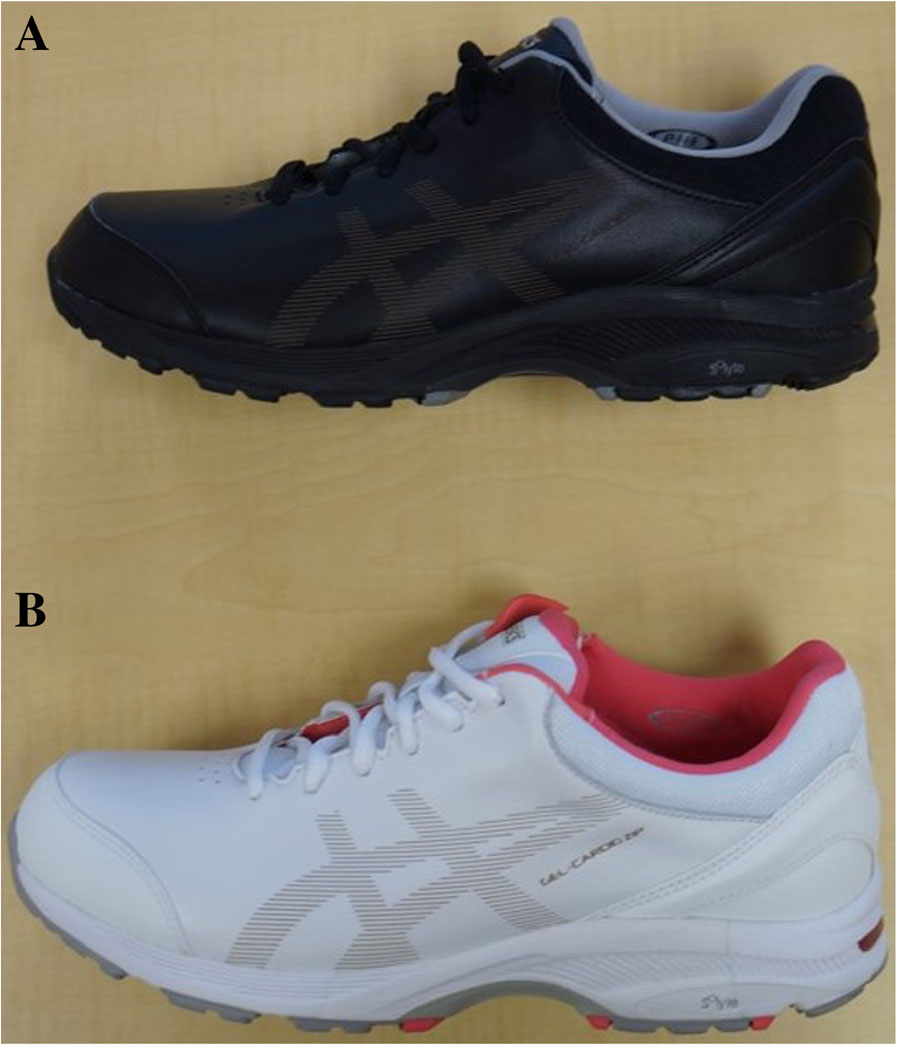
ASICS Cardio Zip 3 shoes. **a** Men’s and women’s colour. **b** Women’s colour only. Women had the option of choosing between the two colours

Participants attended two-monthly visits over a 6-month period. At each study visit, participants received standardised podiatric care comprising of palliative care of nails and skin, temporary padding, wound care, emollient use, footwear advice, foot care advice and gout education delivered by an experienced podiatrist (TM). Gout education was delivered using a pamphlet produced by the New Zealand Ministry of Health including information on the causes of gout, the role of urate in development of gout, pharmacological management, monitoring of serum urate levels and general footwear advice (https://www.health.govt.nz/system/files/documents/topic_sheets/stop_gout_booklet-dec2015.pdf). General footwear advice included information regarding footwear comfort, fit, cushioning, sole and heel height. In addition, participants in the footwear intervention group received a pair of ASICS Cardio Zip 3 shoes to wear during daily activities (Fig. 1). This footwear was chosen based on the findings of a previous feasibility study [8], and its characteristics including heel/forefoot cushioning, dual density midsole, wide-fitting option and a zip for ease of fit. To determine the appropriate footwear size, the participant’s foot length and width were measured by the podiatrist using a Brannock device. Women had the option of choosing between a black or white colour, with men having a black colour only. Footwear was then fitted by the podiatrist.

### Outcomes

Outcome measures were measured at baseline and 2, 4, and 6 months. The primary outcome was participant-reported foot pain, measured using a 100-mm visual analogue scale (VAS) using the anchors of ‘no pain’ (0 mm) to ‘very severe pain’ (100 mm) [4, 8]. Secondary outcome measures included participant-reported overall pain (global representation of pain) using 100-mm VAS, with lower scores indicating less pain, patient global assessment using 100-mm VAS, with lower scores indicating better wellbeing, and activity limitation using the Health Assessment Questionnaire (HAQ-II) [10]. Overall pain, patient global assessment and HAQ-II measures have been endorsed by Outcome Measures in Rheumatology (OMERACT) for use in gout studies [11]. Lower limb function was assessed using the activities of daily living and recreational activities subscales of the Lower Limb Tasks Questionnaire (LLTQ), with higher scores indicating better function [12]. Foot impairment and disability was measured using the Leeds Foot Impact Scale (LFIS), with lower scores indicating less impairment and disability [13]. The LLTQ and LFIS have been used in previous gout studies [8, 14]. Participant perceptions of footwear comfort, fit, ease and weight were each evaluated using 100 mm VAS, with lower scores indicating better comfort, fit, ease and weight. Self-reported footwear daily diaries were used to record footwear use and adverse events measured over the 6 months in the footwear intervention group, returned at each study visit [8]. The characteristics of the footwear worn by the control group were measured at each study visit. Participants were also asked at each visit about whether they had experienced a gout flare since the last study visit.

### Sample size

Initial sample size calculations were based on a previous feasibility study [8]. To detect the minimally important difference of − 15 mm in the foot pain VAS (− 17.2 mm detected in feasibility study, *p* = 0.003) with power 0.80, using the repeated measures model detailed below with a baseline to 6-month correlation of 0.30 would require 52 participants in each group (correlation extrapolated from feasibility study). Using a conservatively estimated loss to follow-up rate of 25% at 6 months, the initial aim was to recruit 140 participants. A protocol amendment containing a revised sample size computation was submitted to and approved by an independent data monitoring committee, due to a decline in recruitment and lower than estimated withdrawal rates. The revision used a new estimated baseline to 6-month correlation of 0.49 in the primary outcome and a new dropout rate, based on the first 38 completions. A revised sample size of 39 completions per group was determined. At an estimated loss to follow-up rate of 15%, the target recruitment was 92 participants (46 per group).

### Statistical analysis

Primary and secondary analyses were based on an intention-to-treat (ITT) analysis set, from which only participants with no baseline nor post-randomisation data were excluded. Primary and other outcomes were compared across the treatment groups using repeated measures models of outcome data at 2, 4, and 6 months adjusted for baseline. A blind review of the data was undertaken at the end of the trial to consider the specific regression models to use, inclusion of covariates, the appropriateness of multiple imputations for any missing covariate and any necessary data transformation. Continuous outcomes were fitted using linear mixed models except for footwear-related visual analogue scale outcomes, fitted with a scaled zero-inflated beta regression model (generalised additive model for location, scale and shape) due to an increased proportion of zero scores recorded for these outcomes. Age, gender, ethnicity, BMI, colchicine use, non-steroidal anti-inflammatory drug use, prednisone use and the presence of subcutaneous tophi at baseline were considered for inclusion in the regression models during a blind review, absent all knowledge of allocation. Partial *R*^2^ was used as the main selection criterion. Ten multiply imputed data sets were produced, using all observed data, under an assumption of Missingness at Random. No data transformation was found to be needed. No correction for multiple testing was applied. All tests were carried out at a significance level of 0.05 against two-sided alternatives. Data were analysed using SAS version 9.4 and R version 3.2.

## Results

### Participant flow and characteristics

**Fig. 2:**
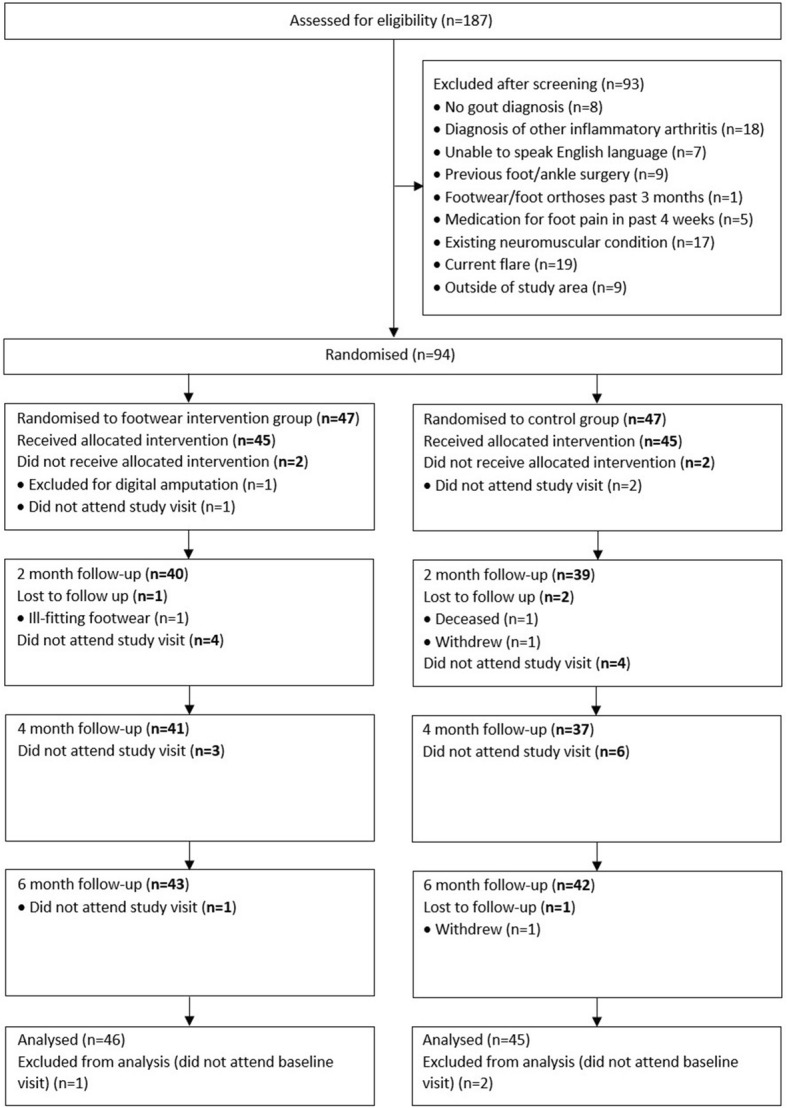
Flow of participants through the study

Figure 2 shows the flow of participants through the study. There were 187 potential participants screened and 94 randomised. Participants were predominantly male of New Zealand European ethnicity, with over 10 year’s disease duration and on urate-lowering therapy (Table 1). High rates of obesity and comorbidities such as hypertension and cardiovascular disease were observed. Notable differences between groups included the higher number of tophi reported in the control group. Poor footwear was common at baseline, with the majority of participants wearing footwear which was worn and over 12 months old (Table 2).

**Table 1:** Baseline descriptive statistics

Variable	Footwear intervention group (*n* = 47)	Control group (*n* = 47)
Sex, male, *n* (%)	40 (85%)	43 (91%)
Age, years	62.6 (17.0)	62.4 (13.7)
BMI, kg/m^2^	30.2 (6.4)	32.0 (7.0)
Ethnicity, *n* (%)		
NZ European	28 (62%)	26 (57%)
Pacific	6 (13%)	11 (23%)
Asian	7 (16%)	5 (11%)
Māori	4 (9%)	4 (9%)
Gout history, mean (SD)		
Disease duration (years)	12.2 (11.2)	13.6 (12.3)
Flares prior 3 months	0.7 (0.9)	0.4 (0.7)
Foot tophus, *n* (%)	9 (19%)	17 (36%)
Any tophus, *n* (%)	13 (28%)	24 (51%)
Serum urate, mmol/L	0.39 (0.13)	0.38 (0.11)
Medications, *n* (%)		
Urate-lowering therapy	33 (72%)	30 (64%)
Colchicine	15 (33%)	17 (36%)
Prednisone	9 (20%)	10 (21%)
NSAID	12 (27%)	13 (29%)
Diuretic	8 (18%)	5 (11%)
Medical History, *n* (%)		
Hypertension	22 (48%)	22 (54%)
Cardiovascular disease	13 (28%)	11 (24%)
Type 2 diabetes	7 (15%)	3 (7%)
Peripheral vascular disease	4 (9%)	3 (7%)
Peripheral neuropathy	3 (7%)	5 (11%)

*BMI* body mass index, *NSAID* non-steroidal anti-inflammatory drug

**Table 2:** Baseline footwear characteristics

Variable	Footwear intervention group (*n* = 47)	Control group (*n* = 47)
Footwear type, *n* (%)		
Good	23 (51%)	23 (51%)
Athletic	5	11
Oxford	10	5
Therapeutic	2	0
Walking	6	7
Moderate	3 (7%)	2 (4%)
Boot	3	2
Poor	19 (42%)	20 (44%)
Sandal	8	4
Moccasin	4	7
Flip-flop	4	4
Slipper	2	4
Court	1	0
Mule	0	1
Footwear age, *n* (%)		
< 6 months	8 (18%)	12 (27%)
6–12 months	5 (11%)	9 (20%)
> 12 months	31 (70%)	24 (53%)
Upper wear, *n* (%)		
Neutral	34 (77%)	26 (58%)
Medial	9 (20%)	17 (38%)
Lateral	1 (2%)	2 (4%)
Midsole wear, *n* (%)		
Neutral	38 (86%)	31 (69%)
Medial	9 (20%)	9 (20%)
Lateral	1 (2%)	5 (11%)
Outsole wear, *n* (%)		
None	2 (4%)	7 (16%)
Partly worn	35 (78%)	29 (64%)
Fully worn	8 (18%)	9 (20%)
Outsole wear pattern, *n* (%)		
None	1 (2%)	6 (13%)
Normal	17 (38%)	19 (42%)
Medial	0 (0%)	0 (0%)
Lateral	27 (60%)	20 (44%)

Recorded protocol violations included randomisation of three participants who consented but did not complete the initial study visit (excluded from the ITT set), and the withdrawal of one participant who consented but was later found to have had foot surgery with a toe amputation, post-randomisation (included in the ITT set).

Four participants in the footwear intervention group were unable to wear their allocated footwear due to discomfort. Of these participants, three remained enrolled in the trial and continued to receive the other facets of their allocated intervention with one participant withdrawal. Participants in the footwear intervention group reported wearing their allocated footwear on average 24 h per week during the study period. Participants in the control group continued to wear footwear of similar type, age and wear during the study period (Additional file 1). At the 6-month follow-up, 89% of the control group and 91% of the footwear intervention group completed the study.

### Primary outcome

All efficacy endpoints and covariate adjustments are shown in Table 3. Baseline foot pain scores were low. There was no difference in foot pain at any time-point over the 6-month study period between the two groups (adjusted effect estimate: − 6.7, 95% CI − 16.4 to 2.9, *P* = 0.17).

**Table 3:** Outcome measure scores and effect estimates adjusted from baseline

	Footwear intervention group mean (SD)	Control group mean (SD)	Adjusted effect estimate	*P*
Foot pain VAS				
Baseline	14.8 (18.7)	17.5 (22.4)		
2 months	10.7 (13.0)	16.8 (21.8)	− 5.0 (− 12.9 to 2.8)	0.21
4 months	13.8 (23.0)	16.1 (22.3)	− 1.8 (− 10.1 to 6.4)	0.66
6 months	13.1 (20.8)	20.5 (26.1)	− 6.7 (− 16.4 to 2.9)	0.17
Overall pain VAS^a^				
Baseline	18.7 (19.6)	17.7 (23.9)		
2 months	9.7 (13.6)	23.3 (27.5)	− 13.2 (− 22.2 to − 4.3)	*< 0.01*
4 months	16.2 (19.4)	17.9 (22.8)	− 2.3 (− 0.5 to 0.6)	0.65
6 months	16.3 (19.2)	20.7 (26.8)	− 4.0 (− 13.6 to 5.7)	0.42
Patient Global Assessment VAS^a^				
Baseline	22.7 (24.5)	21.5 (25.8)		
2 months	17.7 (24.2)	16.4 (21.6)	1.2 (− 7.4 to 9.9)	0.78
4 months	14.6 (16.6)	16.6 (20.2)	− 2.8 (− 11.9 to 6.3)	0.55
6 months	15.3 (19.4)	18.8 (21.9)	− 3.4 (− 12.6 to 5.7)	0.46
Health Assessment Questionnaire II				
Baseline	0.5 (0.6)	0.4 (0.5)		
2 months	0.5 (0.6)	0.4 (0.4)	− 0.1 (− 0.3 to 0.1)	0.36
4 months	0.6 (0.6)	0.3 (0.5)	0.0 (−0.2 to 0.2)	0.84
6 months	0.5 (0.5)	0.4 (0.6)	− 0.1 (− 0.3 to 0.1)	0.28
LFIS total score				
Baseline	15.5 (11.5)	15.4 (12.5)		
2 months	13.8 (13.0)	16.4 (14.1)	− 4.7 (− 9.1 to − 0.3)	*0.04*
4 months	14.9 (14.2)	14.2 (12.3)	− 1.3 (− 6.1 to 3.5)	0.59
6 months	14.4 (13.6)	16.9 (14.2)	− 3.0 (0.2 to 1.8)	0.21
LLTQ activities of daily l				
Baseline	32.7 (8.2)	33.8 (6.8)		
2 months	34.8 (7.2)	32.9 (8.0)	2.2 (− 0.2 to 4.6)	0.07
4 months	32.9 (8.1)	35.4 (6.7)	− 0.4 (− 3.1 to 2.3)	0.77
6 months	34.0 (6.9)	33.8 (7.7)	1.1 (− 1.2 to 3.4)	0.35
LLTQ recreational activities^b^				
Baseline	22.7 (11.8)	21.1 (11.6)		
2 months	23.5 (14.2)	22.8 (11.5)	0.8 (− 2.8 to 4.4)	0.66
4 months	20.8 (12.8)	25.1 (9.9)	− 3.4 (− 7.5 to 0.8)	0.11
6 months	21.7 (12.6)	22.2 (12.0)	− 0.9 (− 4.8 to 3.0)	0.66
Footwear comfort VAS^c^				
Baseline	24.0 (21.9)	27.6 (28.0)		
2 months	10.3 (13.1)	26.2 (26.5)	− 10.4 (− 19.9 to − 0.9)	*0.03*
4 months	9.1 (9.8)	24.0 (21.0)	− 11.3 (− 21.4 to − 1.3)	*0.03*
6 months	17.5 (23.5)	27.9 (28.4)	− 8.0 (− 19.2 to 3.3)	0.16
Footwear fit VAS^d^				
Baseline	20.6 (20.1)	24.0 (27.2)		
2 months	9.8 (16.0)	22.2 (21.3)	− 9.5 (− 17.2 to − 1.8)	*0.02*
4 months	10.3 (13.6)	22.2 (20.4)	− 11.1 (− 19.9 to − 2.4)	*0.01*
6 months	11.9 (20.0)	27.9 (28.4)	− 11.1 (− 21.1 to − 1.0)	*0.03*
Footwear ease VAS^e^				
Baseline	20.9 (23.0)	19.3 (23.8)		
2 months	12.7 (19.1)	26.8 (28.2)	− 9.8 (− 19.4 to − 0.3)	*0.04*
4 months	10.2 (16.6)	23.8 (25.2)	− 12.3 (− 23.0 to − 1.6)	*0.02*
6 months	11.3 (19.7)	27.9 (28.4)	− 13.2 (− 23.8 to − 2.7)	*0.01*
Footwear weight VAS^d^				
Baseline	21.9 (21.9)	22.7 (24.6)		
2 months	12.7 (17.8)	27.0 (26.6)	− 9.7 (− 19.5 to 0.0)	*0.05*
4 months	13.6 (20.3)	24.6 (20.4)	− 10.8 (− 20.6 to − 0.9)	*0.03*
6 months	11.4 (19.7)	27.9 (28.4)	− 10.3 (− 19.8 to − 0.8)	*0.03*

*VAS* visual analogue scale, *LFIS* Leeds Foot Impact Scale, *LLTQ* Lower Limb Tasks Questionnaire

^a^BMI adjusted

^b^Age adjusted

^c^BMI and prednisone adjusted

^d^Sex and BMI adjusted

^e^Ethnicity and BMI adjusted

Data in italics indicates statistical significance

### Secondary outcomes

Improvements between groups in overall pain scores (adjusted effect estimate: − 13.2, 95% CI − 22.2 to − 4.3, *P* < 0.01) favouring the footwear intervention were observed at 2 months, but there was no difference between the groups at 4 or 6 months (adjusted effect estimate at 6 months: − 4.0, 95% CI − 13.6 to 5.7, *P* = 0.42). Foot-related impairment and disability was reduced at 2 months in the footwear intervention group (adjusted effect estimate: − 4.7, 95% CI − 9.7 to − 0.3, *P* = 0.04), but there was no difference between groups at 4 or 6 months (adjusted effect estimate at 6 months: − 3.0, 95% CI 0.2 to − 1.8, *P* = 0.21). No between-group differences in patient global assessment, HAQ-II and LLTQ were observed (Table 2).

Between-group differences favouring the footwear intervention were observed in footwear comfort at 2 months (adjusted effect estimate: − 10.4, 95% CI − 19.9 to − 0.9, *P* = 0.03) and 4 months (adjusted effect estimate: − 11.3, 95% CI − 21.4 to − 1.3, *P* = 0.03), but not at 6 months (adjusted effect estimate: − 8.0, 95% CI − 19.2 to 3.3, *P* = 0.16). Similarly, between-group differences favouring the footwear intervention were observed in footwear fit (adjusted effect estimate: − 11.1, 95% CI − 23.0 to − 1.0, *P* = 0.03), footwear ease (adjusted effect estimate: − 13.2, 95% CI − 23.8 to − 2.7, *P* = 0.01) and footwear weight (adjusted effect estimate: − 10.3, 95% CI − 19.8 to − 0.8, *P* = 0.03) at all time-points over the 6-month study period.

### Adverse events

Two participants (4%) in the footwear intervention group developed foot blisters and one participant (1%) in the footwear intervention group withdrew from the study due to footwear discomfort. During the trial period, 16 participants (34%) in the control group and 14 participants (30%) in the footwear intervention group experienced a gout flare.

## Discussion

This is the first randomised controlled trial of a podiatric intervention in gout. Although improvements in footwear comfort, fit and ease were observed in the footwear intervention group throughout the study period, no significant difference in foot pain was observed between groups. Short-term improvements in both overall pain and foot-related impairment and disability favouring the footwear intervention group were observed at the two-month time-point, consistent with the previous feasibility study [8].

The low levels of foot pain at the time of the baseline visit may have contributed to a floor effect, suggesting that clinical meaningful changes in foot pain could not be detected. Foot pain was not part of the inclusion criteria based on the previous feasibility study [8], which may have also contributed to the baseline foot pain levels observed. This highlights the challenge of studying pain as an outcome in gout which is an intermittently flaring condition. We observed baseline serum urate levels were close to target guidelines [15] and participants reported a low number of flares in the 3 months prior to the trial, which suggests generally well-controlled disease. Our findings for baseline foot pain levels were lower than the previous feasibility study [8], however, were consistent with previous studies measuring foot pain in people with longstanding gout during an intercritical period [4, 15].

Comfort and fit have been identified as important factors in footwear selection for people with gout [7]. Footwear is an important concern for people with gout, who often describe difficulty finding suitable footwear [16]. Improvements in footwear comfort, fit, weight and ease were observed in the footwear intervention group. The footwear received by the footwear intervention group had a number of characteristics which have been identified as beneficial for people with gout when compared to participants own footwear [8]. Footwear characteristics including correct footwear fit, the presence of cushioning and good torsional stiffness have previously been identified as influencers of subjective footwear comfort [17]. In the footwear intervention group, the fitting of footwear by a clinician may also be a factor. The footwear habits of the control group did alter during the trial, despite the footwear advice delivered. This furthers highlight the challenges that people with gout have finding appropriate footwear [16, 18]. These findings suggest that helping people with gout find footwear with good characteristics is important; however, this may not involve the need for expensive footwear prescription.

Strengths of this study include the use of OMERACT-endorsed patient-reported outcomes for gout [11], high retention rates in both groups, and novelty as the first randomised controlled trial of a podiatric intervention in people with gout. The key study limitation was that participants could not be blinded to the footwear intervention, which may have biased the study outcomes, as all end-points were patient-reported. We did attempt to reduce this bias by informing participants that they would be receiving a foot care package without the specific mention of receiving footwear and ensuring that all participants received a comprehensive foot care intervention. The study was undertaken in an urban New Zealand city and the findings may lack generalisability to other settings. The footwear used in this study had distinct characteristics, so it is unclear whether these findings can be generalised to other types of footwear such as open-toed footwear.

This study has focused on a commercially available athletic shoe. The podiatric care package was limited to standardised care and foot health advice, with or without the footwear intervention, and the role of other interventions such as foot orthoses is unknown. Further investigation into other footwear interventions for people with gout, including cost-effectiveness, is warranted. Changes to structural properties of the footwear through use may have also been a potential reason that long-term benefits were not observed. The effects of wear on the structural properties of footwear over time, and its relationship with biomechanical parameters such as plantar pressure and patient-reported outcomes such as foot pain, impairment and disability, are unknown. Future work might also explore factors which influence foot care and footwear use, and the willingness to pay for appropriate footwear.

## Conclusions

The footwear intervention did not significantly improve foot pain in people without high baseline levels of foot pain. However, short-term improvements in overall pain and foot impairment/disability, and more durable improvements in footwear comfort and fit were observed with the footwear intervention.

## Additional file

Additional file 1: Control group footwear characteristics. (DOCX 16 kb)

## Abbreviations

BMI: Body mass index
HAQ-II: Health Assessment Questionnaire
ITT: Intention-to-treat
LFIS: Leeds Foot Impact Scale
LLTQ: Lower Limb Tasks Questionnaire
OMERACT: Outcome Measures in Rheumatology
VAS: visual analogue scale
