# Supplementary material for: Effects of a footwear intervention on foot pain and disability in people with gout: a randomised controlled trial
Source: Arthritis Res Ther. 2019 Apr 24;21:104. doi: 10.1186/s13075-019-1886-y (PMC6480516; doi:10.1186/s13075-019-1886-y)
Supplement: Supplementary file 1 — Control group footwear characteristics. (DOCX 16 kb) [file 13075_2019_1886_MOESM1_ESM.docx]

Additional file 1. Control group footwear characteristics

| **Variable** | **Baseline** | **Two months** | **Four months** | **Six months** |
| --- | --- | --- | --- | --- |
| Footwear type, n (%)  Good  Athletic  Oxford  Therapeutic  Walking  Moderate  Boot  Poor  Sandal  Moccasin  Flip-flop  Slipper  Court  Mule  Heel  Ugg boot | 23 (51%)  11  5  0  7  2 (4%)  2  20 (44%)  4  7  4  4  0  1  0  0 | 17 (44%)  5  7  0  5  4 (10%)  4  18 (46%)  2  9  4  2  1  0  0  0 | 15 (42%)  2  5  0  8  4 (11%)  4  17 (47%)  3  10  3  1  0  0  0  0 | 17 (40%)  5  5  0  7  5 (12%)  5  20 (48%)  3  9  4  2  0  0  1  1 |
| Footwear age, n (%)  <6 months  6-12 months  >12 months | 12 (27%)  9 (20%)  24 (53%) | 10 (26%)  9 (23%)  20 (51%) | 8 (22%)  10 (28%)  18 (50%) | 10 (24%)  8 (19%)  24 (57%) |
| Upper wear, n (%)  Neutral  Medial  Lateral | 26 (58%)  17 (38%)  2 (4%) | 25 (64%)  11 (28%)  3 (8%) | 21 (58%)  13 (36%)  2 (6%) | 24 (57%)  16 (38%)  2 (5%) |
| Midsole wear, n (%)  Neutral  Medial  Lateral | 31 (69%)  9 (20%)  5 (11%) | 30 (77%)  5 (13%)  4 (10%) | 27 (75%)  6 (17%)  3 (8%) | 29 (69%)  11 (26%)  2 (5%) |
| Outsole wear, n (%)  None  Partly worn  Fully worn | 7 (16%)  29 (64%)  9 (20%) | 5 (13%)  30 (77%)  4 (10%) | 3 (8%)  27 (75%)  6 (17%) | 3 (7%)  36 (86%)  3 (7%) |
| Outsole wear pattern, n (%)  None  Normal  Medial  Lateral | 6 (13%)  19 (42%)  0 (0%)  20 (44%) | 4 (10%)  13 (33%)  0 (0%)  22 (56%) | 3 (8%)  15 (42%)  1 (3%)  17 (47%) | 3 (7%)  19 (45%)  18 (43%)  2 (5%) |
